# Leaf Fungal Microbiome Is Modulated by Interspecific Hybridization Events Between *Coffea* Species

**DOI:** 10.1111/ppl.71016

**Published:** 2026-07-08

**Authors:** Leandro Pio de Sousa, Ludmila dos Passos e Silva, Matheus Pena Passos, Juliana Lischka Sampaio Mayer, Marcelo Mendes Brandão, Oliveiro Guerreiro‐Filho, Jorge Maurício Costa Mondego

**Affiliations:** ^1^ Laboratory of Multitrophic Interactions and Biodiversity, Department of Animal Biology, Institute of Biology State University of Campinas Campinas São Paulo Brazil; ^2^ Laboratory of Integrative and Systemic Biology (LaBIS), Center for Molecular Biology and Genetic Engineering State University of Campinas Campinas São Paulo Brazil; ^3^ Laboratory of Plant Anatomy, Department of Plant Biology, Institute of Biology State University of Campinas Campinas São Paulo Brazil; ^4^ Coffee Center Alcides Carvalho Agronomic Institute of Campinas Campinas São Paulo Brazil; ^5^ Laboratory of Biotechnology and Plant‐Microorganism Interaction (LABIPLAM), Center for Plant Genetic Resources Agronomic Institute of Campinas Campinas São Paulo Brazil

**Keywords:** *Coffea*, fungi, interspecific hybrids, leaf, microbiome

## Abstract

Increasing attention has been given to the host phylogeny and domestication roles in shaping plant‐associated microbiomes. However, the interspecific effects of hybridization on microbial communities remain poorly understood. We investigated the effects of interspecific hybridization on the composition, diversity, ecological organization, and co‐occurrence patterns of leaf‐associated fungal communities in five *Coffea* species and hybrids between *C. arabica* and the other four species in the same habitat. Beta‐diversity analyses showed a differentiation among host genotypes. Assignment of fungal genera to guilds indicated that fungal communities were dominated by pathogen–saprotrophs. Interestingly, *Coffea stenophylla* , a genetically distinct species within the same broader evolutionary clade, exhibited a higher relative abundance of pigmented yeasts and saprotrophs compared to *C. arabica* and other *Coffea* species analyzed. Fungal communities associated with hybrids were more similar to those of *C. arabica* than to the other parental species, indicating asymmetric contributions of parental traits to the colonization of the hybrids' phylloplane. A co‐occurrence network revealed that neutral associations were more prevalent in *Coffea* hybrids than in *Coffea* species. These results indicate that while dominant fungal taxa are largely conserved across *Coffea* species and hybrids, interspecific hybridization is associated with the reorganization of the ecological relations in a fungal community. Overall, host genetics and hybridization‐related traits influence the assembly and ecological organization of leaf‐associated fungal communities in *Coffea*.

## 1 Introduction

Coffee is one of the most important commodities in the global market, involving approximately 100 million workers throughout its production chain, particularly in developing countries (ICO 2021). Among the 130 species of the genus *Coffea*, *Coffea arabica* and *Coffea canephora* are the only commercially cultivated species, accounting for 57% and 43% of global coffee production, respectively (ICO 2021). Despite the predominance of *C. arabica* and *C. canephora* , several other *Coffea* species are of agronomic interest due to their beverage quality and tolerance to adverse environmental conditions. For instance, *C. stenophylla* and *C. racemosa* tolerate higher temperatures than *C. arabica* and show increased resistance to pathogens; however, both species exhibit low bean productivity (Sureshkumar et al. 2010; Davis, Mieulet, et al. 2021). Fruit production in coffee plants is determined by a combination of morphological and reproductive traits, including the number of plagiotropic branches, internodes, inflorescences per leaf axil, and flowers per inflorescence. Cultivars of *C. arabica* and *C. canephora* have been selected based on high values for these traits and their interaction with diverse cultivation environments. In contrast, wild, non‐cultivated *Coffea* species typically show reduced fruit production, largely due to fewer numbers of inflorescences per leaf axil and fewer flowers per inflorescence.

Many beneficial traits of wild *Coffea* species can be transferred to *C. arabica* through hybridization (Costa et al. 2025). Notably, *C. arabica* itself originated from a natural hybridization event between the diploid species *C. canephora* and *Coffea eugenioides*, which occurred between approximately 1.08 million and 543,000 years ago (Lashermes et al. 1999). This event resulted in an autogamous allotetraploid species carrying two copies of two distinct genomes (Salojärvi et al. 2024). Autogamy played a crucial role in the speciation of *C. arabica* by preventing the re‐introgression of parental alleles (Lashermes et al. 2014).

Interspecific hybrids among species of the genus *Coffea* can be readily produced and constitute an informative model for investigating the effects of hybridization on plant phenotypes. In studies addressing genetic relationships within the genus, Medina Filho et al. (1984) adapted the gene pool model proposed by Harlan and de Wet (1971), classifying *Coffea* species according to the difficulty of gene transfer to cultivated *C. arabica* . In this framework, *C. canephora* , *C. racemosa* , and *C. eugenioides* were placed in a secondary gene pool with high potential for Arabica coffee improvement, whereas *C. stenophylla* was assigned to the tertiary gene pool due to genetic barriers that limit hybridization. Despite the low fertility of hybrids between *C. arabica* and *C. stenophylla* (Carvalho and Monaco 1967), successful hybrids have been obtained through the coffee breeding program conducted by the Agronomic Institute of Campinas (IAC; Medina‐Filho et al. 2007).

The phyllosphere is defined as the interface between plant surfaces and the atmosphere (Berg et al. 2016) and can harbor more than 10^26^ microorganisms globally (Vorholt 2012), particularly bacteria and fungi (Delmotte et al. 2009). These microorganisms influence plant growth and performance by producing phytohormones (Bisht et al. 2025), protecting hosts against pathogens (Carrión et al. 2019), and enhancing tolerance to abiotic stress (Kaczmarczyk et al. 2011). Consequently, understanding the diversity, composition, and dynamics of phyllosphere microbial communities is highly relevant to microbial ecology. Host plant identity is one of the main factors shaping phyllosphere microbial compositions (Gomes et al. 2018; Whipps et al. 2008). Different plant species harbor distinct microbial communities because leaf morphology and surface properties create specific microhabitats and ecological niches (Hunter et al. 2010). However, there is an ongoing debate regarding the relative importance of deterministic processes driven by host genetics versus stochastic processes in phyllosphere colonization and community assembly (Wagner 2021). From the host perspective, several factors influence microbiome structure, including ploidy level (Wipf and Coleman‐Derr 2021), interspecific variation (Fitzpatrick et al. 2018), plant organ identity (Guo et al. 2021), and developmental stage (Wagner, Busby, and Balint‐Kurti 2020). Domestication and breeding have also been shown to affect microbial diversity, often leading to reduced diversity (Bulgarelli et al. 2015; Coleman‐Derr et al. 2016), although increases in diversity have also been reported (Abdullaeva et al. 2020; Abdelfattah et al. 2022). More recently, attention has focused on how host interspecific hybridization influences the microbiome and how hybrid morphology may modulate these effects (Ding et al. 2022).

In this study, we investigated the fungal communities associated with the leaf surfaces of *C. arabica* hybrids generated with four other *Coffea* species (Data S1) maintained in the germplasm collection of the Agronomic Institute of Campinas. Figure S1 depicts the current natural distributions of these species. We sequenced the fungal internal transcribed spacer (ITS) region to characterize fungal diversity across parental species and hybrids. We focused on the phylloplane fungal microbiome because most diseases affecting coffee leaves are caused by fungal pathogens, including *Hemileia vastatrix*, *Cercospora coffeicola*, and *Phoma* spp., the causal agents of coffee leaf rust, brown eye spot, and Phoma leaf spot, respectively (Santos et al. 2007). Understanding how fungal communities are structured may therefore provide insights into disease control strategies. Notably, leaf surface transplantation from coffee leaf rust–resistant species ( *C. stenophylla* and *C. racemosa* ) to susceptible *C. arabica* plants conferred resistance to *H. vastatrix* infection (de Sousa and Mondego 2024), highlighting the potential of leaf microbiota manipulation for improving plant resistance. The use of a germplasm‐based experimental design allowed us to minimize edaphoclimatic variation, thereby isolating host‐related effects on fungal community assembly.

## 2 Materials and Methods

### 2.1 Collection of Biological Materials

Leaves were collected from adult plants maintained in the Germplasm Bank of the Agronomic Institute of Campinas (IAC), Campinas, São Paulo, Brazil (22°53′ S, 47°05′ W; 664 m a.s.l.), on October 29th, 2024 (spring in the Southern Hemisphere). The sampled plants were located in an open field within a maximum distance of 100 m from each other and therefore shared similar growing conditions, including temperature, irrigation regime, soil type, and management practices. Sampling was conducted at 9:00 a.m. under the following environmental conditions: temperature of 21.7°C, relative humidity of 85%, wind speed of 20.1 km h^−1^, and atmospheric pressure of 1111 hPa. The regional climate is classified as tropical with a dry winter (Aw), according to the Köppen–Geiger climate classification. The soil supporting the germplasm collection is a clayey Oxisol, according to the USDA Soil Taxonomy.

### 2.2 Plant Material

Three individuals of each genotype were selected for sampling, including *C. arabica* , its parental species *C. canephora* and *C. eugenioides*, and hybrids of *C. arabica* with *C. canephora* , *C. eugenioides*, *C. racemosa* , and *C. stenophylla* (further details in Data S1). The hybrids are from the first generation (F_1_ hybrids) of crossings, indicating that likely the proportion of each progenitor species is 50%. From each individual, three plagiotropic branches at 120 cm above the soil were selected, and four fully expanded adult leaves were collected per branch using sterile gloves. The leaves were placed in sterile paper bags for transport and subsequent processing. Half of the collected leaves were used for phylloplane DNA sampling, and the remaining half were used for leaf architectural analyses.

### 2.3 Leaf Anatomical Analyses

Fully expanded leaves from four individuals of each *Coffea* species and hybrids were collected and immediately fixed in 10% neutral buffered formalin (NBF; Boon and Drijver 1986). To ensure adequate tissue penetration, samples were subjected to vacuum infiltration using a vacuum pump (−400 mmHg). After 48 h of fixation, the leaf samples were dehydrated through a graded ethanol series with increasing concentrations and subsequently stored in 70% (v/v) ethyl alcohol until further processing. Transverse sections were obtained from the median region of the leaf blade, equidistant from the base and apex, excluding the midrib. Samples previously stored in 70% ethyl alcohol were further dehydrated through an ethanol series and embedded in plastic resin (Leica Historesin, Leica Microsystems) following the manufacturer's instructions. Cross‐sections with a thickness of 5–7 μm were produced using a manual rotary microtome (Leica Microsystems) equipped with a C‐type steel knife. Sections were stained with 0.05% toluidine blue O (TBO) in phosphate buffer (pH 4.5; Sakai 1973) and mounted in synthetic resin (Entellan, Merck KGaA). Prepared sections were examined under an Olympus BX51 light microscope (Olympus Corporation) coupled to an Olympus DP71 digital camera. Quantitative measurements of anatomical traits were obtained using the ImageJ software (Schneider et al. 2012). The measured traits included mesophyll thickness, the relative contributions of palisade and spongy parenchyma, and the thickness of adaxial and abaxial epidermis tissues. For each individual, measurements were obtained from multiple sections and averaged prior to statistical analyses.

### 2.4 Microbial Collection, DNA Extraction, Sequencing, and Sequence Processing

Microbial cells from the leaf surfaces were collected using sterile cotton‐tipped swabs moistened with a 0.9% NaCl solution. DNA extraction was performed using magnetic beads (MagMax, Thermo Fisher Scientific) following the manufacturer's protocol. Fungal communities were characterized by high‐throughput sequencing of the ITS1 region, amplified using the ITS1 (GAACCWGCGGARGGATCA) and ITS2 (GCTGCGTTCTTCATCGATGC) primers (Schmidt et al. 2013). Sequencing libraries were prepared using TruSeq DNA Sample Preparation Kits (Illumina) and sequenced on an Illumina MiSeq platform with paired‐end reads of 300 nucleotides, using standard Illumina sequencing primers. Raw sequence data were processed using QIIME 2, following the workflow described by Vernier et al. (2020). High‐quality sequences were trimmed and clustered into operational taxonomic units (OTUs) at 97% sequence similarity using Mothur v1.48.0 (https://github.com/mothur/mothur/releases, accessed December 2024). Representative OTU sequences were aligned using PyNAST (Caporaso et al. 2010), and taxonomic assignment was performed using the UNITE database to estimate fungal abundances. All sequencing data have been deposited in the NCBI Sequence Read Archive (SRA) under accession number PRJNA1004833.

### 2.5 Data Analyses

Alpha diversity of the fungal communities was estimated using the Shannon diversity index, calculated with the *folio* package in R. Differences in alpha diversity were assessed using analysis of variance (ANOVA). Community composition and beta diversity were evaluated using permutational multivariate analysis of variance (PERMANOVA) with the adonis2 function in the *vegan* package. Bray–Curtis dissimilarity indices were calculated to compare fungal community composition among all coffee species and hybrids. Differences in fungal community structure were visualized using non‐metric multidimensional scaling (nMDS) based on Bray–Curtis dissimilarities, generated with the *vegan* and *ggplot2* packages in R. A dendrogram representing similarities in mycobiome structure was constructed using the *stats*, *cluster*, and *dendextend* packages in R. The phylogenetic tree of the coffee plants was built using concatenated ITS sequences (Data S2), following the approach described by Anthony et al. (2010). Sequences were retrieved from the National Center for Biotechnology Information (NCBI), aligned using the MUSCLE algorithm, and phylogenetic distances were inferred using the neighbor‐joining method implemented in MEGA12 software. Fungal ecological guilds were inferred using the FUNGuild database, which assigns putative ecological roles to fungal taxa based on curated literature and expert knowledge. Taxonomic assignments at the genus level were used as input, and guild classifications were retrieved using the most recent version of the FUNGuild annotation framework. Only guild assignments with confidence levels classified as “probable” or “highly probable” were retained for downstream analyses, whereas taxa with ambiguous, conflicting, or low‐confidence assignments were excluded. Relative abundances of major guild categories, including pathogen–saprotroph, plant pathogen, saprotroph, and symbiotroph, were calculated for each sample and expressed as proportions of the total annotated fungal community. Guild‐based analyses were used exclusively for comparative and descriptive purposes, providing a coarse ecological framework to complement taxonomic and network‐based analyses, and do not represent direct measurements of fungal functional activity. Fungal co‐occurrence networks were constructed at the genus level. To evaluate the overall effect of hybridization, we pooled *Coffea* species and *Coffea* hybrids into two independent groups and compared their fungal co‐occurrence network interactions. Pearson correlation coefficients between pairs of OTUs were calculated using the *Hmisc* package in R, and correlation matrices were visualized using Cytoscape software. Edge thickness represents the strength of correlations, while blue, red, and gray edges indicate negative, positive, and neutral associations, respectively.

### 2.6 Statistical Analysis

All datasets were evaluated for normality using the Shapiro–Wilk test and Q–Q plots, homogeneity of variances using Levene's test, and the presence of extreme outliers. When assumptions of normality and homoscedasticity were met, one‐way ANOVA followed by Tukey's post hoc test was applied. For normally distributed data with heteroscedasticity, Welch's ANOVA followed by the Games–Howell post hoc test was used. Non‐normally distributed data were analyzed using the Kruskal–Wallis test followed by Dunn's post hoc test. All statistical analyses and graphical visualizations were performed in RStudio (version 2023.06.1; R Core Team 2021) using the *readxl*, *dplyr*, *ggplot2*, *car*, *rstatix*, *FSA*, and *PMCMRplus* packages. To estimate the potential contribution of each parental species to the fungal communities of the hybrids, the SourceTracker2 program was applied. This Bayesian approach estimates the relative contributions of defined source communities to sink communities (Abdelfattah et al. 2022). In this analysis, parental species were treated as potential sources, and each hybrid genotype was defined as a sink.

## 3 Results

### 3.1 Foliar Macroscopic Traits

Macroscopic evaluation of leaf morphology was conducted for the nine *Coffea* genotypes included in this study (Figure S2). Overall, hybrids tended to exhibit leaf shapes more similar to *C. arabica* than to their non‐*arabica* parental species. For example, *C. arabica × C. eugenioides* (AR × EU), *C. arabica × C. stenophylla* (AR × ST), and *C. arabica × C. racemosa* (AR × RA) displayed elliptic‐ovate to oblong leaves with a glossy dark‐green coloration comparable to *C. arabica* . In contrast, *C. arabica × C. canephora* (AR × CA) showed a leaf morphology more similar to *C. canephora* , characterized by a more elongated blade and a tendency for the lamina to bulge between veins, resulting in a crinkled texture (Figure S2).

### 3.2 PCA of Foliar Anatomical Traits

**FIGURE 1:**
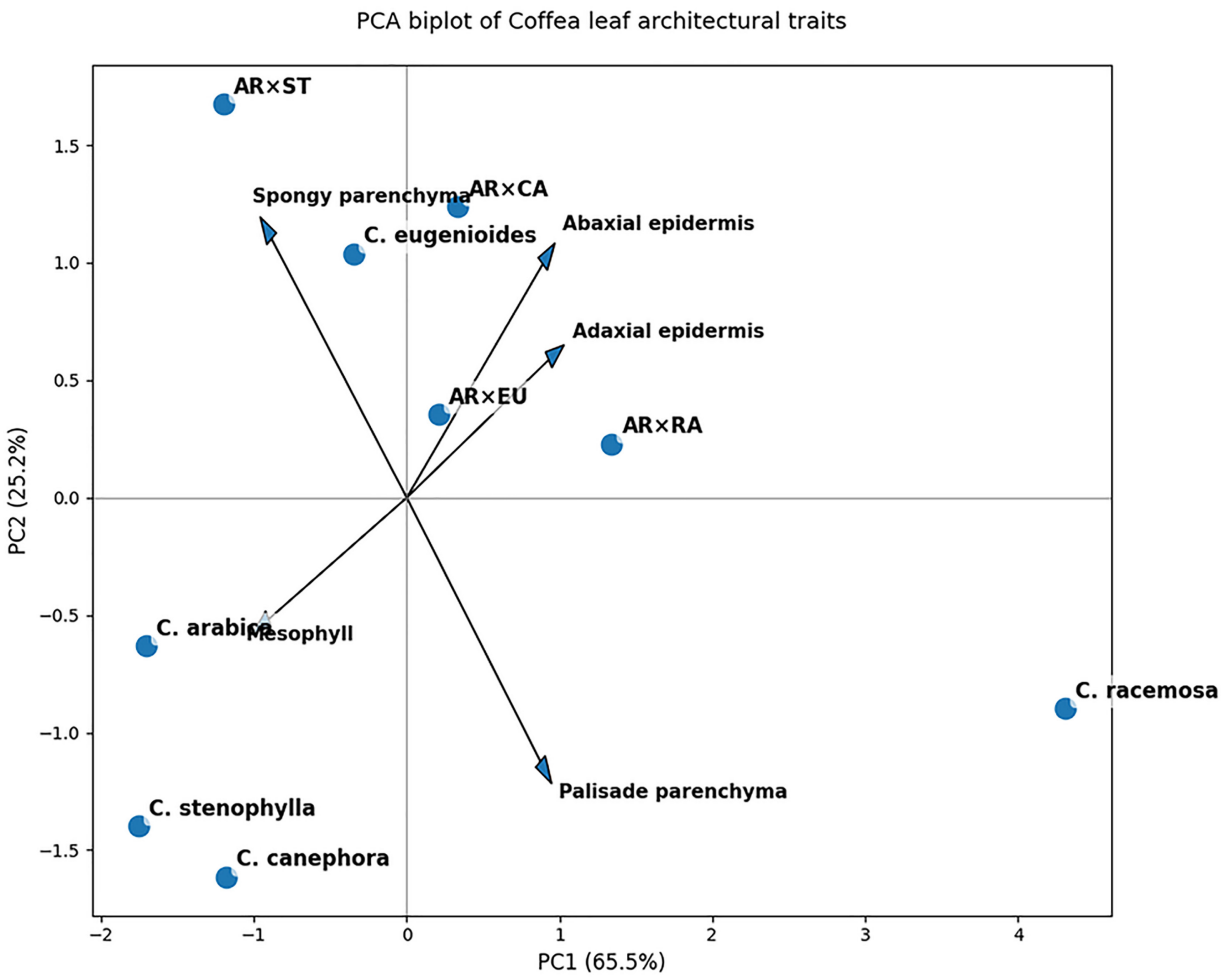
Leaf architectural variation among *Coffea* species and interspecific hybrids. Principal component analysis (PCA) biplot based on quantitative leaf anatomical traits, including mesophyll thickness, relative contributions of palisade and spongy parenchyma, and adaxial and abaxial epidermis thickness. Points represent genotype means calculated from four leaves per genotype, and arrows indicate the direction and relative contribution of each trait to the ordination. The first two axes explain 90.7% of the total variance (PC1 = 65.5%; PC2 = 25.2%).

Quantitative measurements of leaf anatomical traits, including mesophyll thickness, relative contributions of palisade and spongy parenchyma, and adaxial and abaxial epidermis thicknesses, revealed continuous variation in leaf anatomy among *Coffea* species and hybrids (Figures 1 and S3). Principal component analysis (PCA) based on these traits showed that the first two axes explained 90.7% of the total variance (PC1 = 65.5%; PC2 = 25.2%). PC1 represented a composite gradient associated with overall leaf structural investment, with strong contributions from mesophyll thickness, epidermal thicknesses, and the relative proportions of palisade and spongy tissues. PC2 captured finer variation in internal tissue compartmentalization, particularly the balance between palisade and spongy parenchyma. In the PCA ordination, except AR × ST, hybrids generally occupied intermediate positions within the phenotypic space defined by the parental species, whereas some genotypes exhibited more distinct anatomical profiles along the major axes (Figure 1). These results, together with foliar macroscopic traits, provide a quantitative anatomical framework for contextualizing differences observed among genotypes in subsequent mycobiome analyses.

### 3.3 Sequencing Overview and Fungal Diversity

**FIGURE 2:**
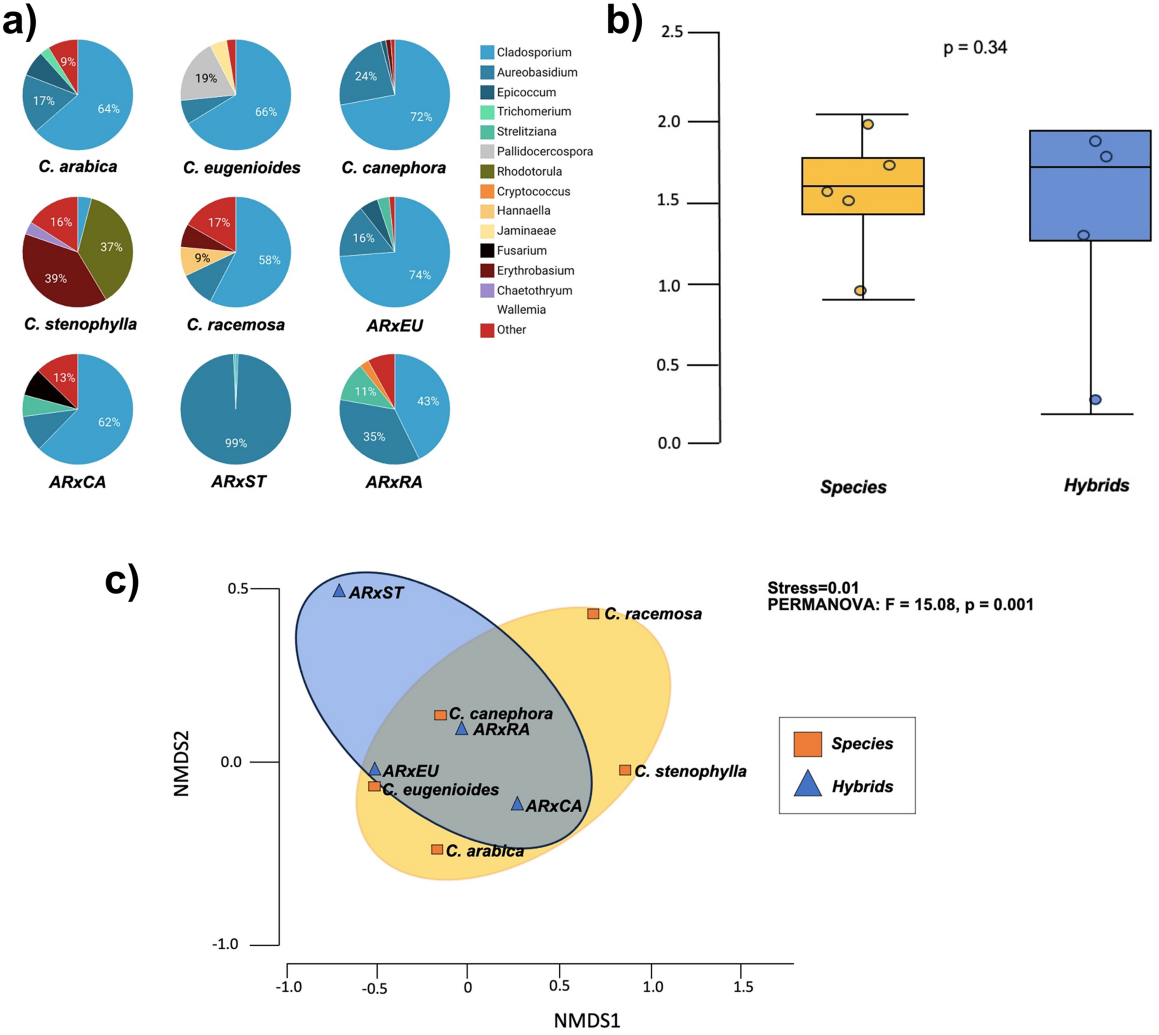
(a) Phyllosphere fungal community composition at the genus level; (b) Alpha diversity (Shannon indices) was analyzed using a repeated measures two‐way ANOVA; (c) Beta diversity analyzed using non‐metric multidimensional scaling (nMDS) plot based on Bray–Curtis dissimilarity between samples of the leaves mycobiome. The closer the points, the more similar of microbial community composition. The ellipses represent the standard deviation of data points belonging to each group (species vs. hybrids). PERMANOVA was used to evaluate intercommunity significance.

To characterize fungal diversity in the leaf phylloplane, ITS1 metabarcoding was performed using DNA collected from the leaf surfaces of the nine *Coffea* genotypes. A total of 523,602 high‐quality fungal sequences were obtained and clustered into 736 operational taxonomic units (OTUs). Fungi belonging to the phylum Ascomycota accounted for more than 95% of the detected OTUs, followed by Basidiomycota. At the class level, Dothideomycetes was the most abundant group, followed by Eurotiomycetes. At the genus level, 95 fungal genera were identified. *Aureobasidium* and *Cladosporium* were the only genera detected in all parental species and hybrids (Figures 2a and S4). Other detected genera included *Pallidocercospora*, *Strelitziana*, and *Epicoccum* (Figures 2a and S4).

No significant differences in alpha diversity were detected between parental species and hybrids (Mann–Whitney *U* = 13.0, *p* = 0.34), although effect size was moderate‐to‐large (*r* = 0.30; Cohen's *d* = 0.75), suggesting that the absence of significance likely reflects limited statistical power (*n* = 3 per genotype) rather than a true absence of biological effect (Figure 2b).

Beta‐diversity analyses revealed significant differences in community composition (Figure 2c). Among the hybrids, AR × ST exhibited the greatest compositional dissimilarity relative to both parental species, demonstrating a distinct fungal community profile from all other genotypes examined. Conversely, AR × EU clustered closely with *C. arabica* and *C. eugenioides* in the PCA graph, indicating high similarity in community composition to both parental species. Intermediate patterns were observed for AR × CA and AR × RA, which displayed moderate dissimilarity relative to *C. canephora* and *C. racemosa* , respectively (Figure 2c). Homogeneity of multivariate dispersion was confirmed prior to PERMANOVA interpretation (Betadisper test—Levene test on distances to group centroid: *F* = 0.0004, *p* = 0.98; Cohen's *d* = 0.06), indicating that the observed differences in community composition reflect true location differences in multivariate space rather than differences in within‐group dispersion.

### 3.4 Structural Comparison of Mycobiomes Among Species and Hybrids

**FIGURE 3:**
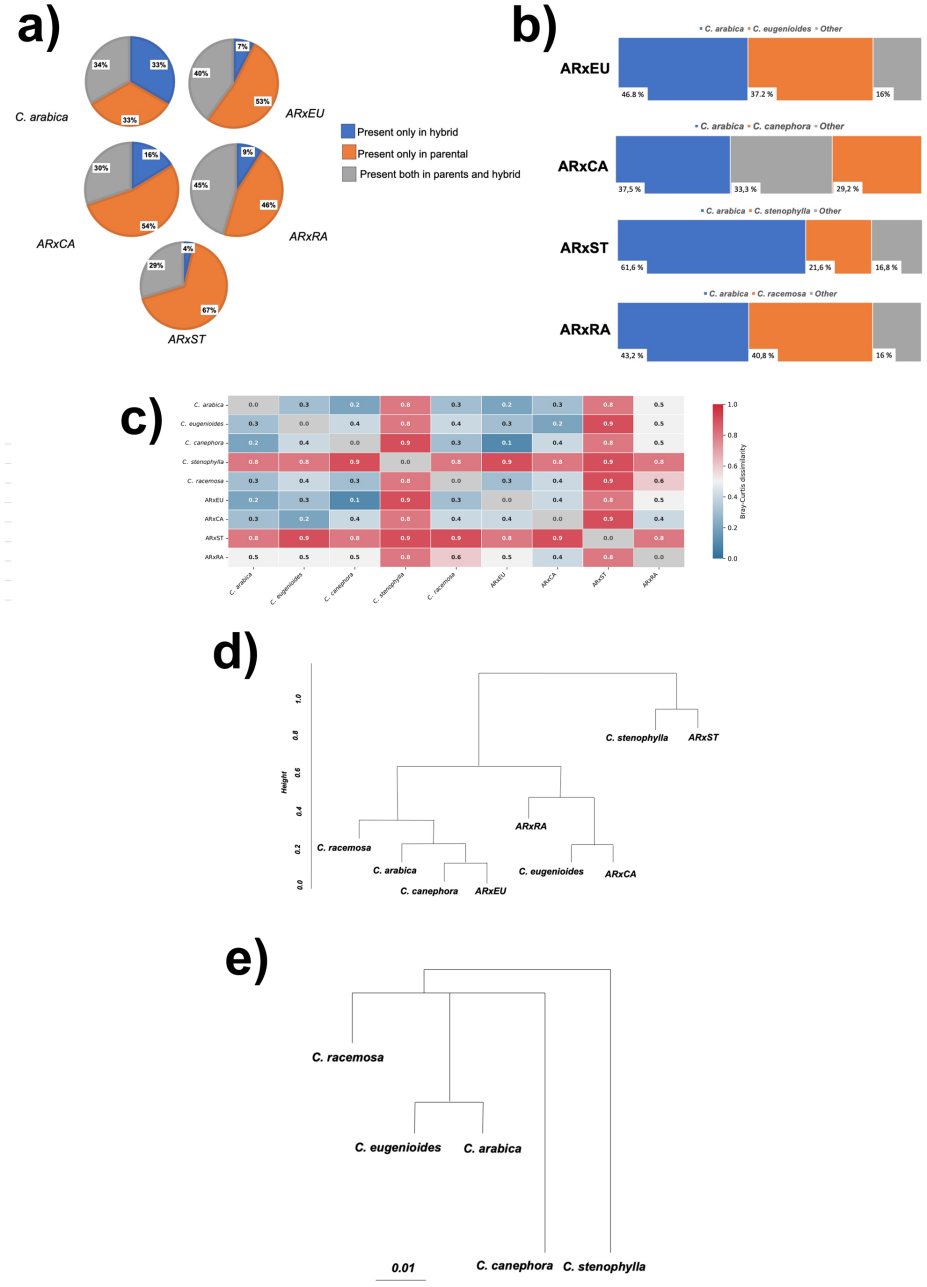
Structural comparison of fungal communities among *Coffea* species and interspecific hybrids. (a) Proportion of shared and exclusive fungal genera among parental species and hybrids. (b) Estimated contribution of parental mycobiomes to hybrid fungal communities inferred using SourceTracker. (c) Heatmap of Bray–Curtis dissimilarities among fungal communities. The redder the greater the dissimilarity; the bluer the lower the dissimilarity (d) Dendrogram representing fungal community similarity. (e) Phylogenetic relationships among *Coffea* species based on concatenated ITS sequences.

Comparison of fungal community composition revealed that, relative to its parental species *C. canephora* and *C. eugenioides*, 34% of the fungal genera detected were exclusive to *C. arabica* (Figure 3a). Hybrids exhibited between 4% and 16% of fungal genera that were unique to themselves and absent from both parental species. Among hybrids, AR × CA showed the highest proportion of exclusive genera, whereas AR × ST showed the lowest proportion (Figure 3a). Estimates of parental contributions to hybrid mycobiomes, visualized using treemap representations (Figure 3b), indicated a predominance of the *C. arabica* fungal community in all hybrids, with estimated contributions ranging from 37.5% to 61.6%. Heatmap visualization of Bray–Curtis dissimilarities among mycobiomes (Figure 3c) and related dendrogram analysis (Figure 3d) suggested greater dissimilarity between *C. arabica* and *C. stenophylla* , as well as between *C. stenophylla* and its hybrid with *C. arabica* . We inferred a phylogenetic tree using concatenated ITS sequences and used it as a proxy for genetic distance among *Coffea* species (Figure 3e; Data S2). Interestingly, this pattern was somewhat similar to that observed in the mycobiome analyses in which *C. stenophylla* and its hybrid formed distinct groups relative to other species and hybrids (Figure 3e).

### 3.5 Shifts in Fungal Abundance Distributions Between Parental Species and Hybrids

**FIGURE 4:**
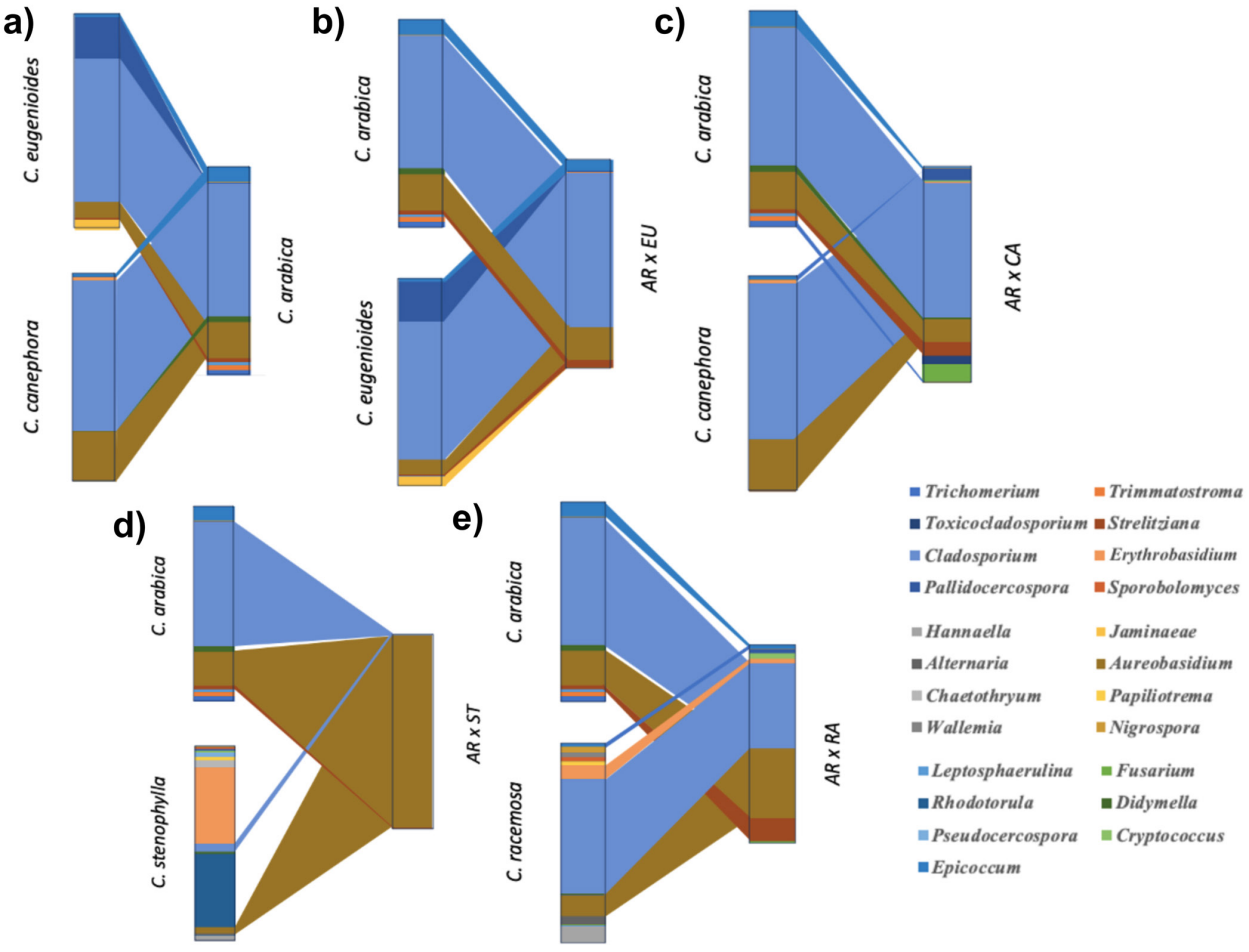
Modified Sankey diagram showing changes in fungal abundance distributions between *Coffea* parental species and interspecific hybrids. Modified Sankey diagram illustrating the relative distribution of fungal genera across *Coffea* parental species and their respective interspecific hybrids. The width of the flows is proportional to the relative abundance of each genus, highlighting shifts in dominant taxa between parental species and hybrids. (a) Parental species: *C. canephora* × *C. eugenioides*, interspecific hybrid: *C. arabica* ; (b) Parental species: *C. eugenioides* × *C. arabica* , interspecific hybrid: AR × EU; (c) Parental species: *C. canephora* × *C. arabica* , interspecific hybrid: AR × CA; (d) Parental species: *C. stenophylla* × *C. arabica* , interspecific hybrid: AR × ST; (e) Parental species: *C. racemosa* × *C. arabica* , interspecific hybrid: AR × RA.

Modified Sankey diagrams illustrating relative genus abundances (Figure 4) visually summarized changes in fungal community composition between parental *Coffea* species and their interspecific hybrids. Both parental species and hybrids were generally dominated by *Cladosporium*, followed by *Aureobasidium*. An exception was observed for AR × ST, in which *Aureobasidium* represented the dominant genus (Figure 4). The Sankey representation highlights redistribution patterns of dominant fungal taxa between parental species and hybrids without implying direct inheritance or interaction mechanisms.

### 3.6 Distribution of Fungal Ecological Guilds

**FIGURE 5:**
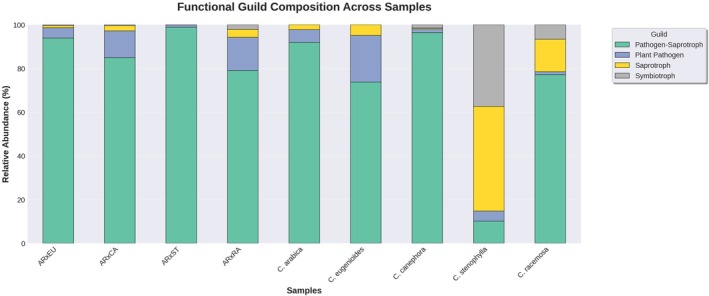
Ecological guild composition of fungal communities associated with *Coffea* species and hybrids. Stacked bar plots show the relative abundance (%) of major fungal ecological guilds inferred using FUNGuild, including pathotroph–saprotrophs, plant pathogens, saprotrophs, and symbiotrophs. Guild assignments represent putative ecological roles and were used for comparative purposes.

Assignment of fungal genera to ecological guilds using FUNGuild revealed that fungal communities associated with *Coffea* species and hybrids were dominated by taxa classified as pathogen–saprotrophs across all genotypes (Figure 5). This guild accounted for the largest proportion of the community in both parental species and hybrids, generally exceeding 75% of the relative abundance. Despite this overall dominance, marked differences in the relative contributions of secondary guilds were observed among genotypes. Plant pathogens represented a variable fraction of the fungal communities, ranging from low proportions in *C. stenophylla* and *C. canephora* to higher relative abundances in *C. eugenioides* and in some hybrids, particularly AR × CA and AR × RA (Figure 5). In contrast, saprotrophic fungi showed substantial variation among parental species. Notably, *C. stenophylla* exhibited a markedly higher proportion of saprotrophs compared to the other genotypes, whereas saprotrophs represented a minor component of the fungal communities associated with *C. arabica* and *C. canephora* . Intermediate proportions of saprotrophs were observed in several hybrids. The symbiotroph guild was consistently detected at low relative abundances across most genotypes but showed a pronounced increase in *C. stenophylla* . The hybrid involving *C. stenophylla* displayed lower proportions of symbiotrophs compared to the parental species, suggesting a shift in guild composition in the hybrid phyllosphere.

### 3.7 Fungal Co‐Occurrence Network Patterns

**FIGURE 6:**
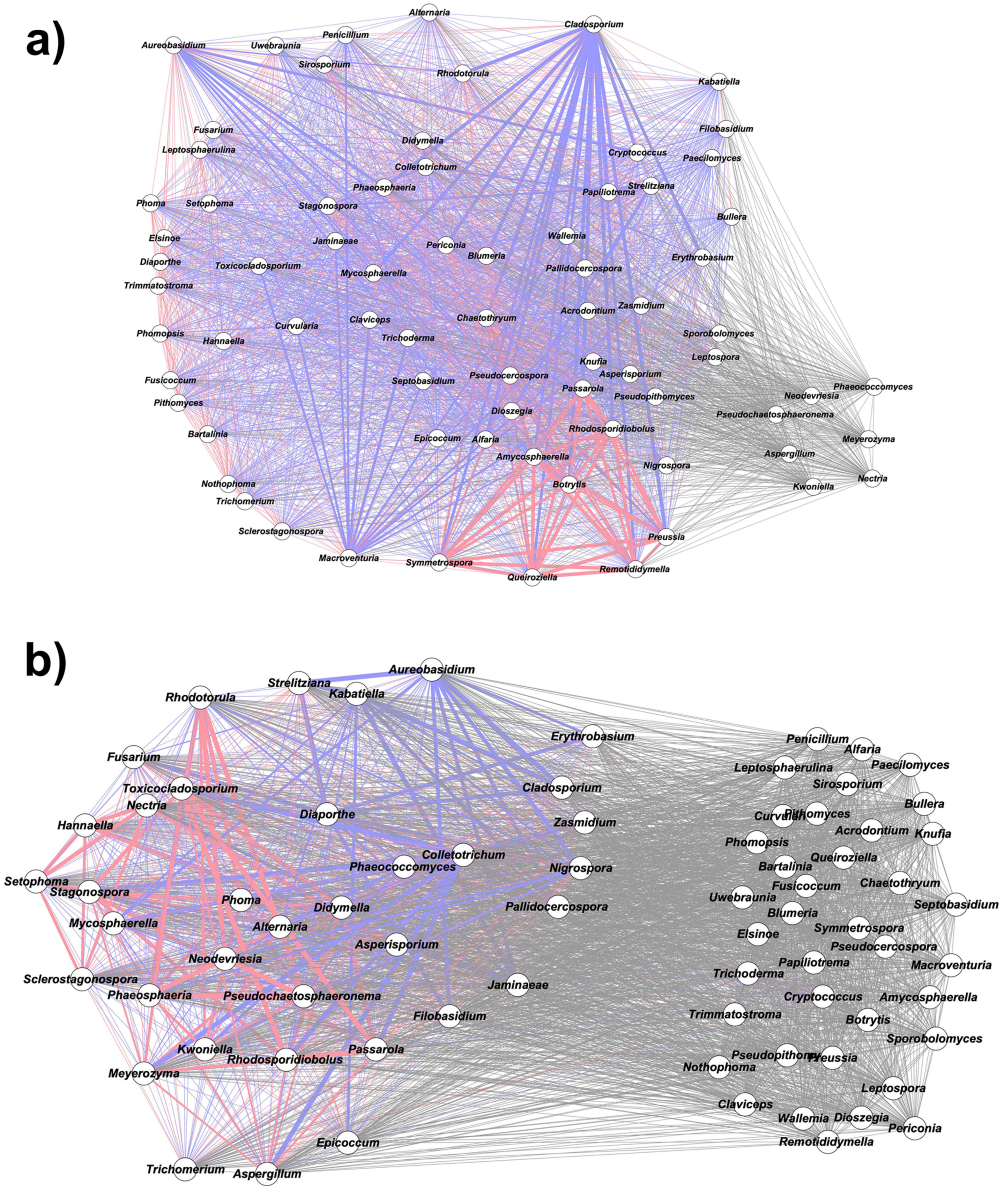
Co‐occurrence network patterns of fungal genera associated with *Coffea* species and interspecific hybrids. Co‐occurrence networks constructed at the genus level based on correlation analyses for parental species (a) and hybrids (b). Nodes represent fungal genera, and edges represent positive (red), negative (blue), or neutral (gray) associations. Networks were used for exploratory and comparative purposes to visualize differences in fungal community organization.

Co‐occurrence network analyses were used to explore patterns of association among fungal genera in parental *Coffea* species and interspecific hybrids (Figure 6). These networks are graph‐based models that represent entities—in our case, fungal genera—as nodes and their statistically valid associations as connecting edges (Barberán et al. 2012). Networks were constructed at the genus level based on correlation analyses and were used for comparative and exploratory purposes. In parental *Coffea* species, a total of 1016 positive, 3038 negative, and 1422 neutral associations were detected among fungal genera. In contrast, fungal communities associated with hybrids exhibited 468 positive, 776 negative, and 4232 neutral associations. Although the absolute numbers of associations differed between the parental and hybrid networks, comparisons focused on relative patterns of association rather than network size or density. Parental networks were characterized by a higher relative proportion of negative associations, whereas hybrid networks were dominated by neutral associations (Figure 6). Within parental networks, genera such as *Cladosporium*, *Aureobasidium*, *Macroventuria*, *Queiroziella*, *Passarola*, and *Botrytis* showed high connectivity. In hybrid networks, highly connected genera included *Aureobasidium*, *Rhodotorula*, *Passarola*, and *Colletotrichum*, contributing substantially to the observed co‐occurrence patterns.

## 4 Discussion

This study investigated how interspecific hybridization within the genus *Coffea* is associated with variation in leaf‐associated fungal communities. In contrast to patterns reported for several domesticated plant systems, in which wild relatives often harbor higher microbial diversity than cultivated genotypes (Porter and Sachs 2020; Favela et al. 2021), no significant differences in alpha diversity were observed between *Coffea* parental species and hybrids (Figure 2b). This suggests that hybridization per se does not strongly affect overall fungal richness in the phylloplane, at least under the uniform edaphoclimatic conditions examined here. Although the proportion of fungal genera detected exclusively in hybrids was relatively small (4%–16%), *C. arabica* itself exhibited a high proportion of exclusive genera (33%).

*C. arabica* is an autogamous allotetraploid species resulting from a natural hybridization event between *C. canephora* and *C. eugenioides* (Bawin et al. 2021; Gomez et al. 2010). The higher number of exclusive fungal genera detected in *C. arabica* may reflect long‐term host–microbe associations that became established during the evolutionary history of this species. While speculative, this pattern raises the possibility that host diversification and stabilization over evolutionary timescales may be accompanied by the gradual differentiation of associated fungal communities—a process that may not yet be fully expressed in recently generated hybrids.

In contrast to alpha diversity, clearer differences were detected in beta diversity among *Coffea* species and hybrids (Figure 2c). In particular, *C. stenophylla* , *C. racemosa* , and the hybrid *C. arabica × C. stenophylla* (AR × ST) showed marked differentiation in fungal community composition. Dissimilarity analyses and heatmap visualizations indicated that *C. stenophylla* and its hybrid were consistently distant from the remaining *Coffea* genotypes. This pattern broadly mirrored host phylogenetic relationships, although hybrids did not systematically cluster with both parental species. Notably, the fungal communities associated with hybrids were generally more similar to those of *C. arabica* than to those of the non‐*arabica* parental species. *SourceTracker* analyses further supported a predominance of the *C. arabica* mycobiome in hybrids (Figure 3), suggesting asymmetric contributions of parental fungal communities. In general, our data point toward the dominance of the *C. arabica* genomic/phenotypic background. For instance, hybrids consistently clustered closer to *C. arabica* , which contributes to 37%–62% of the mycobiome in all hybrids.

One possible explanation for this asymmetry relates to differences in host genomic architecture. *C. arabica* is a polyploid, whereas the other parental species included in this study are diploids. Polyploidy has been proposed as a factor influencing plant‐associated microbiomes, potentially through changes in anatomy, physiology, or chemical traits (Wipf and Coleman‐Derr 2021). In this context, the tendency of hybrids to resemble *C. arabica* in foliar anatomical traits (Figure S2), may provide a structural backdrop favoring fungal communities more similar to those associated with *C. arabica* . However, microscopic analysis of the leaves showed a more complex scenario, wherein, curiously, hybrids seemed to possess intermediate traits compared to *Coffea* species (Figures 1 and S3).

Across all genotypes, the core leaf‐associated mycobiome was composed of only two genera, *Cladosporium* and *Aureobasidium*, which dominated the fungal communities in most parental species and hybrids. Such low taxonomic turnover is consistent with previous reports showing that microbial differentiation among plant species is often weaker in leaves than in belowground compartments (Brown et al. 2021; Wagner, Busby, and Balint‐Kurti 2020; Wagner, Roberts, et al. 2020). The widespread prevalence of these genera across diverse hosts suggests that phylloplane colonization may be governed by strong environmental filtering, favoring taxa that are particularly well‐adapted to the physicochemical conditions of the leaf surface.

*Cladosporium* is a cosmopolitan genus encompassing more than 500 species and is frequently detected as a saprophyte, endophyte, or commensal in a wide range of plant hosts (Bensch et al. 2012; de Sousa et al. 2021). Members of this genus include both plant pathogens and antagonists capable of producing antifungal compounds and acting as biocontrol agents (Wang et al. 2013; Islam 2022; González‐Pérez and Jiménez‐Bremont 2024). In *Coffea*, *Cladosporium* has been reported as an endophyte, a component of the bean microbiome, and a frequent antagonist of *Hemileia vastatrix*, the causal agent of coffee leaf rust (Santamaría and Bayman 2005; Silva et al. 2008; Pereira et al. 2024). In the present study, *Cladosporium* was the most abundant genus in nearly all genotypes, with the notable exception of *C. stenophylla* and its hybrid with *C. arabica* .

*Aureobasidium* was the second most prevalent genus and is likewise known for its cosmopolitan distribution and tolerance to environmental extremes, including osmotic stress, pH variation, and temperature fluctuations (Wang et al. 2022). Beyond its biotechnological relevance, *Aureobasidium* has recently attracted attention as a potential plant growth‐promoting and biocontrol agent (Rensink et al. 2024). The consistent detection of *Aureobasidium* and *Cladosporium* across *Coffea* genotypes—as well as in the phyllosphere of other plant species such as wheat, grapevine, tobacco, olive, and oak (Jumpponen and Jones 2010; Grube et al. 2011; Karlsson et al. 2014; Abdelfattah et al. 2015; Xiang et al. 2022) supports the view that these taxa represent broadly adapted phylloplane colonizers rather than host‐specific specialists.

The case of *C. stenophylla* is particularly noteworthy, as this species and the *C. arabica* × *C. stenophylla* hybrid (AR × ST) deviated from patterns observed in other *Coffea* species and hybrids. *C. stenophylla* is genetically more distant from *C. arabica* and historically more difficult to intercross (Carvalho and Monaco 1967), a factor that may influence microbiome assembly. Its leaf‐associated fungal community was characterized by a high relative abundance of saprotrophs compared to the other genotypes (Figure 5). Wu et al. (2025) detected that rhizospheric soil of *C. arabica* disease‐resistant varieties was enriched with saprotrophic fungi. The presence of these organic matter decomposers can be important for competing with soil pathogens for nutrients. Indeed, the transplantation of *C. stenophylla* leaf microbiota suppressed the development of *H. vastatrix* in susceptible *C. arabica* (de Sousa and Mondego 2024). Additionally, pigmented yeasts such as *Rhodotorula* and *Erythrobasidium* were prevalent in *C. stenophyla*. These two genera produce carotenoid pigments that confer tolerance to oxidative stress and high light or UV exposure (Avalos and Carmen Limón 2015; Gouka et al. 2022), traits that may be advantageous in the phylloplane, particularly under drier and sunnier conditions to which *C. stenophylla* is adapted (Davis et al. 2020; Davis, Gargiulo, et al. 2021). It is possible that other factors, such as differences in leaf surface chemistry or carbohydrate composition, may contribute to the predominance of these yeasts and saprobes, even though such hypothesis remains to be tested.

While taxonomic composition showed substantial stability across genotypes, fungal co‐occurrence networks suggested differences between the parental species and hybrids (Figure 6). In both parental species and hybrids, negative associations were more frequent than positive ones, suggesting that competitive interactions may be prevalent in the phylloplane environment. In hybrids, interestingly, neutral associations accounted for a much larger proportion of total associations compared to parental species. This shift towards neutrality may reflect a reorganization of fungal communities under altered host conditions, potentially associated with hybrid genomic and/or physiological instability (Wipf and Coleman‐Derr 2021; Trivedi et al. 2022). Curiously, microscopic leaf traits of the hybrids were intermediate compared to those of the parental species, perhaps pinpointing a connection between the hybrids' anatomical features, genomic instability, and neutral fungal interactions. This interpretation remains indirect and should be viewed as a working hypothesis rather than a causal explanation.

Network topology also differed between the parental species and hybrids. In parental species, *Cladosporium*, *Aureobasidium*, and *Macroventuria* emerged as highly connected taxa, whereas in the hybrids, *Aureobasidium* remained central while *Cladosporium* lost prominence and *Colletotrichum* became more influential. Additionally, a group of positively associated genera observed in parental species was disrupted in hybrids (Figure 6), suggesting that hybridization alters patterns of fungal co‐occurrence. We hypothesize that hybridization may affect the organization of fungal communities more strongly than their taxonomic composition.

It must be acknowledged that due to limited sample availability, our data rely on three individuals per genotype. While this sample size may be considered small for ITS‐based diversity, network inference, and SourceTracker analyses, and could constrain general inferences, our results strongly suggested that the host genetic background plays a key role in structuring leaf‐associated fungal communities in *Coffea*. The prevalence of dominant fungal genera remained largely conserved across parental species and hybrids. Interestingly, SourceTracker, beta‐diversity, and macroscopic trait analyses indicate a dominance of *C. arabica* traits in the hybrids' mycobiomes. Conversely, network analyses suggest that hybridization affected community organization—particularly co‐occurrence patterns—more than taxonomic richness, shifting toward neutral fungal associations. Both additive/dominant parental effects and emergent hybrid effects support the view that phylloplane colonization by fungi is not a purely stochastic process, and that host traits related to genomic composition, anatomy, and environmental adaptation modulate leaf microbial assembly. From an applied perspective, these findings highlight the relevance of considering microbiome organization, alongside taxonomic composition, in coffee breeding programs aimed at improving resilience to environmental stress.

## Author Contributions

L.P.S. and J.M.C.M. conceived the ideas and designed the experimental methods. L.P.S. and O.G.‐F. collected the plant material. L.P.S., L.P.S., and M.M.B. performed data analysis. M.P.P. and J.L.S.M. performed leaf anatomy analyses. L.P.S. and L.P.S. edited the figures. O.G.‐F. was responsible for coffee germplasm maintenance. L.P.S. and J.M.C.M. wrote the final version of the manuscript.

## Funding

This work was supported by São Paulo Research Foundation (FAPESP, Brazil), Coordination for the Improvement of Higher Education Personnel (CAPES, Brazil), and National Council for Scientific and Technological Development (CNPq, Brazil).

## Conflicts of Interest

The authors declare no conflicts of interest.

## Supporting information


**Figure S1:** Map of coffee tree occurrence in Africa.
**Figure S2:** Diagram showing the leaves of the parental Coffea (*C. arabica,* *C. canephora*, *C. eugenioides*, *C. racemosa* and *C. canephora*) and interspecific hybrids.
**Figure S3:** Anatomical analysis of leaf blades of *Coffea* genotypes. Transverse sections (7 μm thickness) were stained with toluidine blue O in phosphate buffer, and leaf traits were measured (for more details, see material and methods). abe, abaxial epidermis; ade, adaxial epidermis; bs, bundle sheath; is, intercellular spaces; ph, phloem; pp, palisade parenchyma; sp, spongy parenchyma; vb, vascular bundle; xy, xylem. Scale bars: A, C–J = 100 μm; B = 50 μm.
**Figure S4:** Venn diagrams and list of fungal genera present in Coffea and hybrids.
**Data S1:** List of *Coffea* varieties and their hybrids used.
**Data S2:** Concatenated sequences of *Coffea* species according to Anthony et al. (2010). atpB‐rbcL: sequence comprising the intergenic space between *atpB* gene and *rbcL* gene. trnL‐trnF: sequence comprising the trnL gene (which includes an intron) and the intergenic spacer between *trnL* gene and *trnF* gene. trnT‐trnL: sequence comprising the intergenic space between the trnT gene and trnL gene.

## Data Availability

The data that support the findings of this study are openly available in the NCBI Sequence Read Archive (SRA) under accession number PRJNA1004833.
