# Supplementary material for: Leaf Fungal Microbiome Is Modulated by Interspecific Hybridization Events Between Coffea Species
Source: Physiol Plant. 2026 Jul 8;178(4):e71016. doi: 10.1111/ppl.71016 (PMC13345752; doi:10.1111/ppl.71016)
Supplement: Supplementary file 1 — Figure S1: Map of coffee tree occurrence in Africa. Figure S2: Diagram showing the leaves of the parental Coffea (C. arabica, C. canephora, C. eugenioides, C. racemosa and C. canephora) and interspecific hybrids. Figure S3: Anatomical analysis of leaf blades of Coffea genotypes. Transverse sections (7 μm thickness) were stained with toluidine blue O in phosphate buffer, and leaf traits were measured (for more details, see material and methods). abe, abaxial epidermis; ade, adaxial epidermis; bs, bundle sheath; is, intercellular spaces; ph, phloem; pp, palisade parenchyma; sp, spongy parenchyma; vb, vascular bundle; xy, xylem. Scale bars: A, C–J = 100 μm; B = 50 μm. Figure S4: Venn diagrams and list of fungal genera present in Coffea and hybrids. Data S1: List of Coffea varieties and their hybrids used. Data S2: Concatenated sequences of Coffea species according to Anthony et al. (2010). atpB‐rbcL: sequence comprising the intergenic space between atpB gene and rbcL gene. trnL‐trnF: sequence comprising the trnL gene (which includes an intron) and the intergenic spacer between trnL gene and trnF gene. trnT‐trnL: sequence comprising the intergenic space between the trnT gene and trnL gene. [file PPL-178-e71016-s001.docx]

**Supplementary Files**

**Figure S1. Map of coffee tree occurrence in Africa.**

**
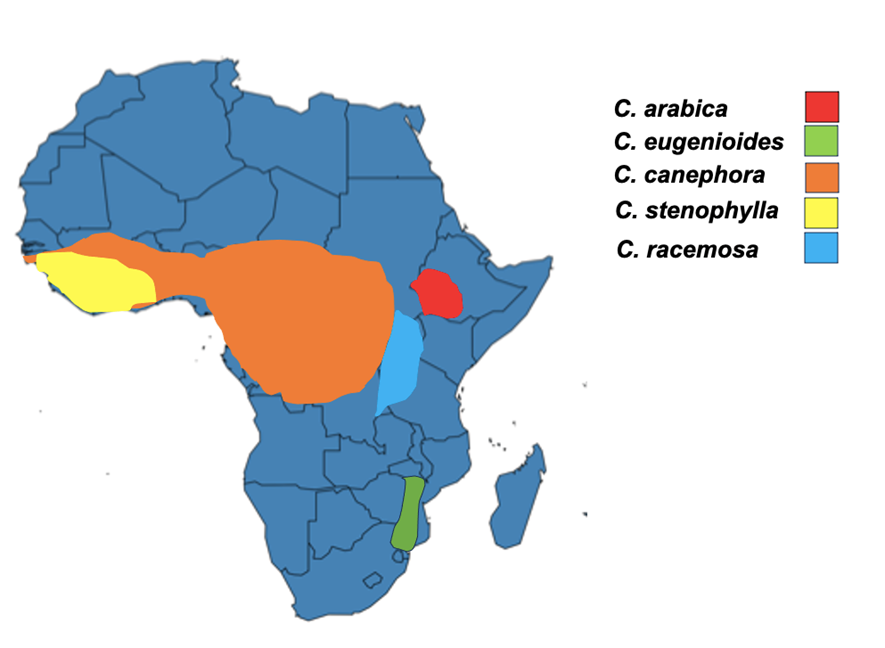
**

**Data S1. List of *Coffea* varieties and their hybrids used**

**Species**:

*Coffea racemosa* H6611-2, 1193-3-2-1, Col.10

*Coffea stenophylla* 1090, 1070-13-1, 1070-13

*Coffea canephora* cv. Conilon Vitória, cv. Robusta

*Coffea arabica* Catuaí Amarelo IAC 62

*Coffea eugenioides*

**Hybrids**:

ARxRA -> *C. arabica* cv. Mundo Novo IAC 387-17 x *C. racemosa* IAC 1195-5 [Hybrid H3640-1; crossing carried out in1956]

ARxST -> *C. arabica* cv. Catuaí Vermelho IAC 81 x *C. stenophylla* IAC 1090 [Hybrid H14296-1; crossing carried out in 1984]

ARxCA -> *C. arabica* cv. Catuaí Vermelho IAC 81 x *C. canephora* cv. Guarini IAC 1598 Dp [Hybrid H14493-1; crossing carried out in 1986]; *C. arabica* cv. Catuaí Vermelho IAC 81 x *C. canephora* cv. Robusta IAC 1330 Dp [Hybrid H14941-1; crossing carried out in 1986]

ARxEU -> *C. arabica* var. typica IAC 10 x *C. eugenioides* IAC 1098-6 1090 [Hybrid H3497-1; crossing carried out in 1956]; *C. arabica* var. laurina IAC 1097-18 x *C. eugenioides* IAC 1098-5 [Hybrid H3423-1; crossing carried out in 1955]

**Figure S2. Diagram showing the leaves of the parental *Coffea* (*C. arabica*, *C. canephora*, *C. eugenioides*, *C. racemosa* and *C. canephora*) and interspecific hybrids.**


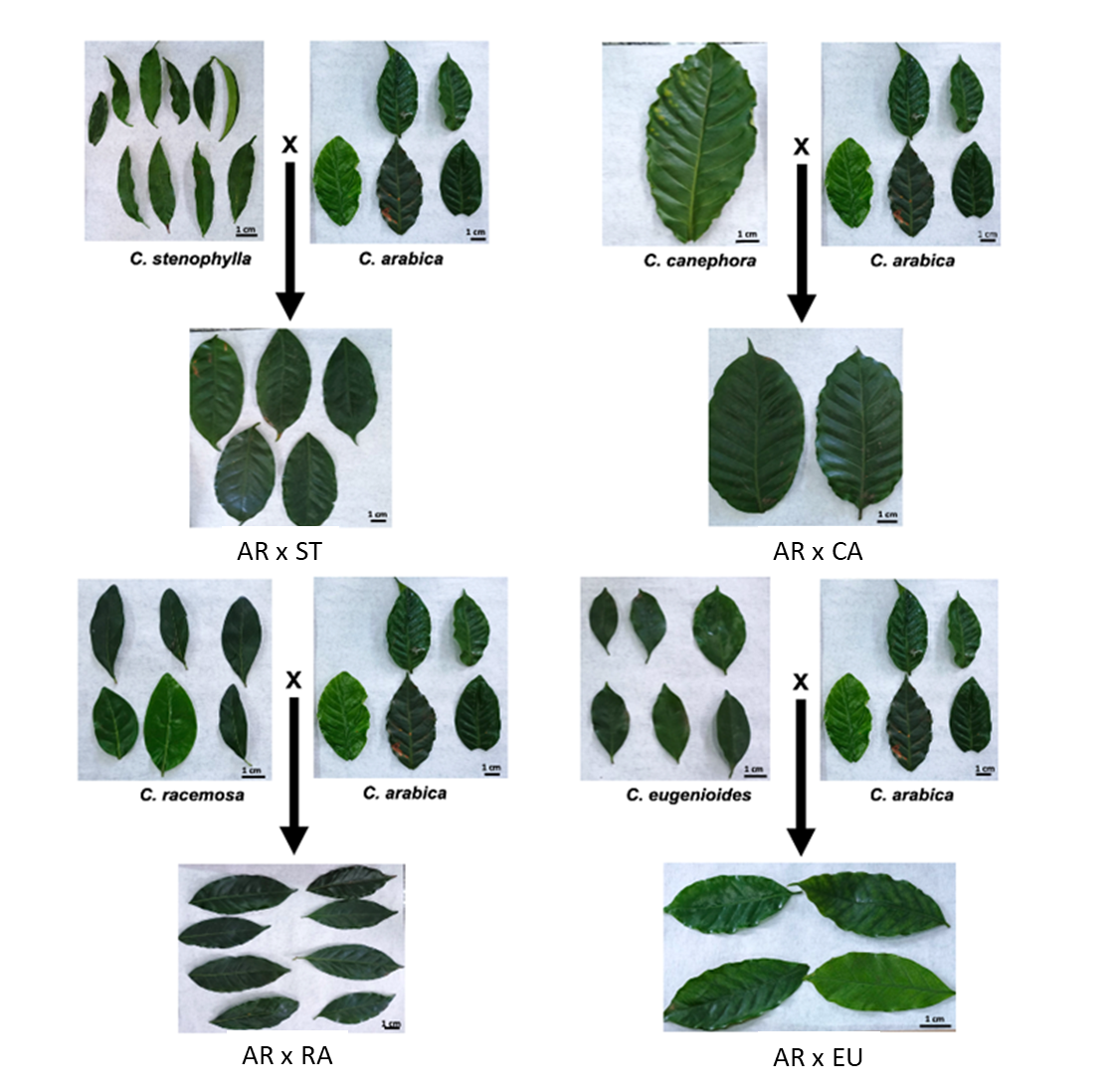


**Data S1. Concatenated sequences of *Coffea* species according to Anthony et al (2010**). *atpB-rbcL*: sequence comprising the intergenic space between *atpB* gene and *rbcL* gene. *trnL-trnF*: sequence comprising the *trnL* gene (which includes an intron) and the intergenic spacer between *trnL* gene and *trnF* gene. *trnT-trnL:* sequence comprising the intergenic space between the *trnT* gene and *trnL* gene.

| **Species** | ***atpB-rbcL*** | ***trnL-trnF*** | ***trnT-trnL*** |
| --- | --- | --- | --- |
| *C. arabica* | FJ493347 | FJ493319 | FJ493374 |
| *C. canephora* | FJ493349 | FJ493321 | FJ493376 |
| *C. eugenioides* | FJ493353 | FJ493326 | FJ493380 |
| *C. racemosa* | FJ493361 | FJ493334 | FJ493388 |
| *C. stenophylla* | FJ493364 | FJ493337 | FJ493391 |

*Coffea arabica*

>CATTTGGTTGGTACCATTCAATCGAATCCAACTCAATTGTTTCTTTATTCAAACAATGAATTTAAATTCAATCAATCTATTTTATTTTTAGAATTTCAAGTGGATGAATAGGAATGTTGAGAAGGTCTTTCATTTATCTATCATTATAGATAATCCCATCTCTATTATCCATTCTATGGAATTCGAACCTGAACTCTATTTACATTACGATTCATTATTTCTATCCCATTGGATTTCTTTTTTATTTCAGCATATCGATTTACGCCGAGCCTTTTTTTCATTTTTTCTTTTTTTATACCTATATCACGAATTCTGTACATTTTCACATCTAGGATTTACATATACAACATATAGCGCTGTCAAGGGGAATTTCTTATTAGTTAGTTATTTTGATTCCAAAGGGGTAAAAAAGAAAATTTGGGTTGCGCTATATATATGAAAGAGTATACAATAATGATGTATTTGGCAAATCGCATGATCTGGTCTAATAATAATAATCAAACATTCTGATTAGTTGATAATATTAGTATTAGTTGGGAATTTTGTGAAAGATTCTTGTGAAAAGTTTCATTAACAGCTAATTCGTGTCGAGTAGACCTTGTTGTTGTGAGAATTCTTAATTCATGAGTTGTAGGGAGGGATTTATGTCACCACAAACAGAGACTAAAGCAAGTGTTGGATTCAATCCCCCAACTATTTATCCTATCCCCCTTTCGTTAGCGGTTCAAAAAACCTTATTCATTTACTCTATTCTCTTAGAAATCGATCTGGACGGAAAAGCCCTTTTCTTATCACAAATCTTGTGTTATTTATGATATACATATAAATGAACATCTTTGAGCAAGAAATACCCATTTGAATGGTTTACAATCGATATAACTATTCATACTGAAACTTACAAAGTACTCTTTTTTAAGATACAAGAAATTCTAGTACCTAGATAAAATTTTGTAATCCCCTTTCCTTCTTTTAATTGACATAGCCCCCCTTTTCTCATAAAATGAGGATGCTACATTGGGACTGGTCGGGATAGCTCAGATGATCTAGAAAAGAATAAAATAGAATTTCAAATAAATTATTGAATATTATAGAGCACAACGATTAATATAGCGATATAGAATTTCGATTTTTTTATCACTCGATTAAATTCGAATTATCATCTACTATTAGATAGTAAATAGTTTTAGTATAGTTAAATTAGTTAAATTTTTCATTTTTGAATTCAAATGACATTTGGAATTCTTTTTTTATACTTCTATATAATTTGATTCTATATCATATATTTCTAATTCTAATTAGGAGTTCTAACTAATGAGACATTCTCCGTTTTCATTCATAAAGATGTAATTAATGTTGTAATGTAAAGTAAATAAATCGTAAAGGCGAAAATTAAGACGACAAAAAAAAGAATCGACCGTTCAACTATTCAAAATTGCATTGGAAAGCTGACAGGGAGATATATATATCTAAGATATATATTCATCTATATTGAATTGGGGATACAGAAATGATAAAATCATATTGGATTGAA

*Coffea canephora*

>CATTTGGTTGGTACCATTCAATCGAATCCAACTCAATTGTTTCTTTATTCAAACAATGAATTTAAATTCAATCAATCTATTTTATTTTTAGAATTTCAAGTGGATGAATAGGAATGTTGAGAAGGTCTTTCATTTATCTATCATTATAGATAATCCCATCTCTATTATCCATTCTATGGAATTCGCTATGGAATTCGAACCTGAACTCTATTTACATTACGATTCATTATTTCTATCCCATTGGATTTCTTTTTGATTTCAGCATATCGATTTACGCCGATCCTTTTTTTCATTTTTTCTTTTTTTATACCTATATCACGAATTCTGTACATTTTCACATCTAGGATTTACATATACAACATATAGCGCTGTCAAGGGGAATTTCTTATTAGTTAGTTATTTTGATTCCAAAGGGGGTAAAAAAGAAAATTTGGGTTGCGCTATATATATGAAAGAGTATACAATAATGATGTATTTGGCAAATCGCATGGTCTGGTCTAATAATCAAACATTCTGATTAGTTGATAATATTAGTATTAGTTGGGAATTTTGTGAAAGATTCTTGTGAAAAGTTTCATTAACAGCTAATTCGTGTCGAGTAGACCTTGTTGTTGTGAGAATTCTTAATTCATGAGTTGTAGGGAGGGATTTATGTCACCACAAACAGAGACTAAAGCAAGTGTTGGATTCAATCCCCCAACTATTTATCCTATCCCCCTTTCGTTAGCGGTTCAAAAAACCTTATTCATTTACTCTATTCTCTTAGAAATCGATCTGGACGGAAAAGCTCTTTTCTTATCACAAATCTTGTGTTATTTATGATATACATATAAATGAACATCTTTGAGCAAGAAATACCCATTTGAATGGTTTACAATCGATATAACTATTCATACTGAAACTTACAAAGTACTCTTTTTTAAGATCCAAGAAATTCTAGTACCTAGATAAAACTTTGTAATCCCCTTTCCGTCTTTTAATTGACATAGCCCCCCTTTTCTCATAAAATGAGGATGCTACATTGGGACTGGTCGGGATAGCTCAGATGATCTAGAAAAGAATAAAATAGAATTTCAAATAAATTATTGAATATTATAGAGCACAACGATTAATATAGCGATATAGAATTTCGATTTTTTTATCACTCGATTAAATTCGAATTATCATCTACTATTAGATAGTAAATAGTTTTAGTATAGTTAAATTAGTTAAATTTTTCATTTTTGAATTCAAATGACATTTGGAATTCTTTTTTTATATTTGATTCTATATCATATATTTCTAATTCTAATTAGGAGTTCTAACTAATGAGACATTCTCCGTTTTCATTCATAAAGATGTAATTAATGTTGTAATGTAAAGTAAATAAATCGTAAAGGCGAAAATTAAGACGACAAAAAAAAGAATCGACCGTTCAACTATTCAAAATTGCATTGGAAAGCTGACAGGGAGATATATATATCTAAGATATATATTCATCTATATTGAATTGGGGATACAGAAATGATAAAATCATATTGGATTGAA

*Coffea eugenioides*

>CATTTGGTTGGTACCATTCAATCGAATCCAACTCAATTGTTTCTTTATTCAAACAATGAATTTAAATTCAATCAATCTATTTTATTTTTAGAATTTCAAGTGGATGAATAGGAATGTTGAGAAGGTCTTTCATTTATCTATCATTATAGATAATCCCATCTCTATTATCCATTCTATGGAATTCGAACCTGAACTCTATTTACATTACGATTCATTATTTCTATCCCATTGGATTTCTTTTTTATTTCAGCATATCGATTTACGCCGAGCCTTTTTTTCATTTTTTCTTTTTTTATACCTATATCACGAATTCTGTACATTTTCACATCTAGGATTTACATATACAACATATAGCGCTGTCAAGGGGAATTTCTTATTAGTTAGTTATTTTGATTCCAAAGGGGTAAAAAAGAAAATTTGGGTTGCGCTATATATATGAAAGAGTATACAATAATGATGTATTTGGCAAATCGCATGATCTGGTCTAATAATAATAATCAAACATTCTGATTAGTTGATAATATTAGTATTAGTTGGGAATTTTGTGAAAGATTCTTGTGAAAAGTTTCATTAACAGCTAATTCGTGTCGAGTAGACCTTGTTGTTGTGAGAATTCTTAATTCATGAGTTGTAGGGAGGGATTTATGTCACCACAAACAGAGACTAAAGCAAGTGTTGGATTCAATCCCCCAACTATTTATCCTATCCCCCTTTCGTTAGCGGTTCAAAAAACCTTATTCATTTACTCTATTCTCTTAGAAATCGATCTGGACGGAAAAGCCCTTTTCTTATCACAAATCTTGTGTTATTTATGATATACATATAAATGAACATCTTTGAGCAAGAAATACCCATTTGAATGGTTTACAATCGATATAACTATTCATACTGAAACTTACAAAGTACTCTTTTTTAAGATACAAGAAATTCTAGTACCTAGATAAAATTTTGTAATCCCCTTTCCTTCTTTTAATTGACATAGCCCCCCTTTTCTCATAAAATGAGGATGCTACATTGGGACTGGTCGGGATAGCTCAGATGATCTAGAAAAGAATAAAATAGAATTTCAAATAAATTATTGAATATTATAGAGCACAACGATTAATATAGCGATATAGAATTTCGATTTTTTTATCACTCGATTAAATTCGAATTATCATCTACTATTAGATAGTAAATAGTTTTAGTATAGTTAAATTAGTTAAATTTTTCATTTTTGAATTCAAATGACATTTGGAATTCTTTTTTTATACTTCTATATAATTTGATTCTATATCATATATTTCTAATTCTAATTAGGAGTTCTAACTAATGAGACATTCTCCGTTTTCATTCATAAAGATGTAATTAATGTTGTAATGTAAAGTAAATAAATCGTAAAGGCGAAAATTAAGACGACAAAAAAAAGAATCGACCGTTCAACTATTCAAAATTGCATTGGAAAGCTGACAGGGAGATATATATATCTAAGATATATATTCATCTATATTGAATTGGGGATACAGAAATGATAAAATCATATTGGATTGAA

*Coffea racemosa*

>CATTTGGTTGGTACCATTCAATCGAATCCAACTCAATTGTTTCTTTATTCAAACAATGAATTTAAATTCAATCAATCTATTTTATTTTTAGAATTTCAAGTGGATGAATAGGAATGTTGAGAAGATCTTTCATTTATCTATCATTATAGATAATCCCATCTCTATTATCCATTCTATGGAATTCGCTATGGAATTCGAACCTGAACTCTATTTACATTACGATTCATTATTTCTATCCCATTGGATTTCTTTTTTATTTCAGCATATCGATTTACGCCGAGCCTTTTTTTCATTTTTTCATTTTTTCATTTTTTCATTTTTTCATTTTTTCTTTTTTTATACCTATATCACGAATTCTGTACATTTTCACATCTAGGATTTACATATACAACATATAGCGCTGTCAAGGGGAATTTCTTATTAGTTAGTTATTTTGATTCCAAAGGGGGTAAAAAAGAAAATTTGGGTTGCGCTATATATATGAAAGAGTATACAATAATGATGTATTTGGCAAATCGCATGGTCTGGTCTAATAATCAAACATTCTGATTAGTTGATAATATTAGTATTAGTTGGGAATTTTGTGAAAGATTCTTATGAAAAGTTTCATTAACAGCTAATTCGTGTCGAGTAGACCTTGTTGTTGTGAGAATTCTTAATTCATGAGTTGTAGGGAGGGATTTATGTCACCACAAACAGAGACTAAAGCAAGTGTTGGATTCAATCCCCCAACTATTTATCCTATCCCCCTTTCGTTAGCGGTTCAAAAAACCTTATTCATTTACTCTATTCTCTTAGAAATCGATCTGGACGGAAAAGCCCTTTTCTTATCACAAATCTTGTGTTATTTATGATATACATATAAATGAACATCTTTGAGCAAGAAATACCCATTTGAATGGTTTACAATCGATATAACTATTCATACTGAAACTTACAAAGTACTCTTTTTTAAGATACAAGAAATTCTAGTACCTAGATAAAACTTTGTAATCCCCTTTCCTTCTTTTAATTGACATAGCCCCCCTTTTCTCATAAAATGAGGATGCTACATTGGGACTGGTCGGGATAGCTCAGATGATCTAGAAAAGAATAAAATAGAATTTCAAATAAATTATTGAATATTATAGAGCACAACGATTAATATAGCGATATAGAATTTCGATTTTTTATCACTCGATTAAATTCGAATTATCATCTACTATTAGATAGTAAATAGTTTTAGTATAGTTAAATTAGTTAAATTTTTCATTTTTGAATTCAAATGACATTTGGAATTCTTTTTTTATACTTCTATATAATTTGATTCTATATCATATATTTCTAATTCTAATTAGGAGTTCTAACTAATGAGACATTCTCCGTTTTCATTCATAAAGATGTAATTAATGTTGTAATGTAAAGTAAATAAATCGTAAAGGCGAAAATTAAGACGACAAAAAAAAGAATCGACCGTTCAACTATTCAAAATTGCATTGGAAAGCTGACAGGGAGATATATATATCTAAGATATATATTCATCTATATTGAATTGGGGATACAGAAATGATAAAATCATATTGGATTGAA

*Coffea stenophylla*

>CATTTGGTTGGTACCATTCAATCGAATCCAACTCAATTGTTTCTTTATTCAAACAATGAATTTAAATTCAATCAATCTATTTTATTTTTAGAATTTCAAGTGGATGAATAGGAATGTTGAGAAGGTCTTTCATTTATCTATCATTATAGATAATCCCATCTCTATTATCCATTCTATGGAATTCGCTATGGAATTCGAACCTGAACTCTATTTACATTACGATTCATTATTTCTATCCCATTGGATTTCTTTTTTATTTCAGCATATCGATTTACGCCGAGCCTTTTTTTCATTTTTTCTTTTTTTATACCTATATCACGAATTCTGTACATTTTCACATCTAGGATTTACATATACAACATATAGCGCTGTCAAGGGGAATTTCTTATTAGTTAGTTATTTTGATTCTAAAGGGGGTAAAAAAGAAAATTTGGGTTGCGCTATATATATGAAAGAGTATACAATAATGATGTATTTGGCAAATCGCATGGTCTGGTCTAATAATCAAACATTCTGATTAGTTGATAATATTAGTATTAGTTGGGAATTTTGTGAAAGATTCTTGTGAAAAGTTTCATTAACAGCTAATTCGTGTCGAGTAGACCTTGTTGTTGTGAGAATTCTTAATTCATGAGTTGTAGGGAGGGATTTATGTCACCACAAACAGAGACTAAAGCAAGTGTTGGATTCAATCCCTCAACTATTTATCCTATCCCCCTTTCGTTAGCGGTTCAAAAAACCTTATTCATTTATTCTATTCTCTTATAAATCGATCTGGACGGAAAAGCCCTTTTCTTATCACAAATCTTGTGTTATTTATGATATACATATAAATGAACATCTTTGAGCAAGAAATACCCATTTGAATGGTTTACAATCGATATAACTATTCATACTGAAACTCACAAAGTACTCTTTTTTAAGATACAAGAAATTCTAGTACCTAGATAAAACTTTGTAATCCCCTTTCCTTCTTTTAATTGACATAGCCCCCCTTTTCTCATAAAATGAGGATGCTACATTGGGACTGGTCGGGATAGCTCAGATGATCTAGAAAAGAATAAAATAGAATTTCAAATAAATTATTGAATATTATAGAGCACAACGATTAATATAGCGATATAGAATTTCGATTTTTTTATCACTCGATTAAATTCGAATTATCATCTACTATTAGATAGTAAATAGTTTTAGTATAGTTAAATTAGTTAAATTTTTCATTTTTGAATTCAAATGACATTTGGAATTCTTTTTTTATACTTCTATATAATTTGATTCTATATCATATATTTCTAATTCTAATTAGGAGTTCTAACTAATGAGACATTCTCCGTTTTCATTCATAAAGATGTAATTAATGTTGTAATGTAAAGTAAATAAATCGTAAAGGCGAAAATTAAGACGACAAAAAAAAGAATCGACCGTTCAACTATTCAAAATTTCATTGGAAAGCTGACAGGGAGATATATATATCTAAGATATATATTCATCTATATTGAATTGGGGATACAGAAATGATAAAATCATATTGGATTGAA

**
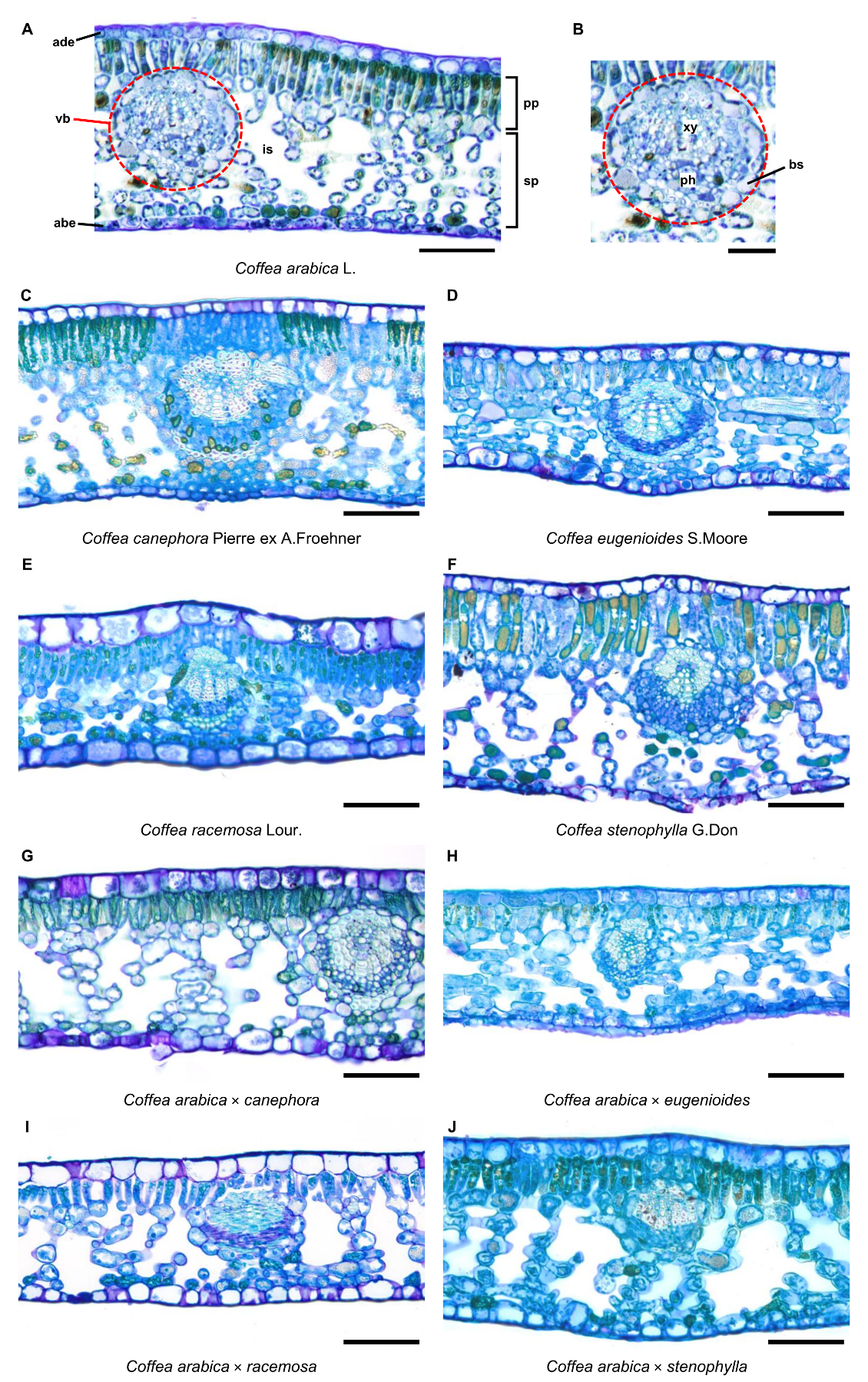
**

**Figure S3: Anatomical analysis of leaf blades of *Coffea* genotypes.** Transverse sections (7 μm thickness) were stained with toluidine blue O in phosphate buffer, and leaf traits were measured (for more details, see material and methods). Abbreviations: abe: abaxial epidermis, ade: adaxial epidermis, bs: bundle sheath, is: intercellular spaces, ph: phloem, pp: palisade parenchyma, sp: spongy parenchyma, vb: vascular bundle, xy: xylem. Scale bars: A, C–⁠J = 100 μm; B = 50 μm.


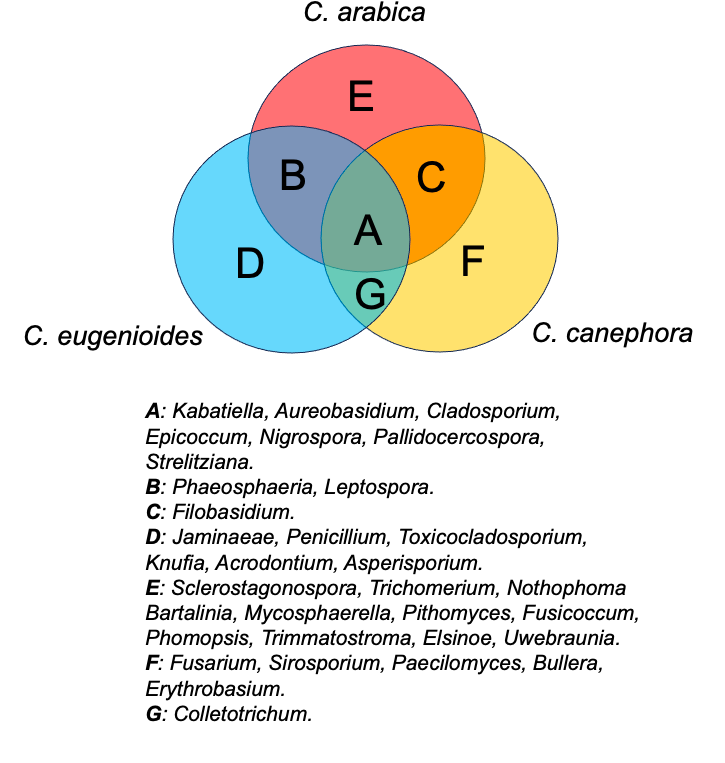


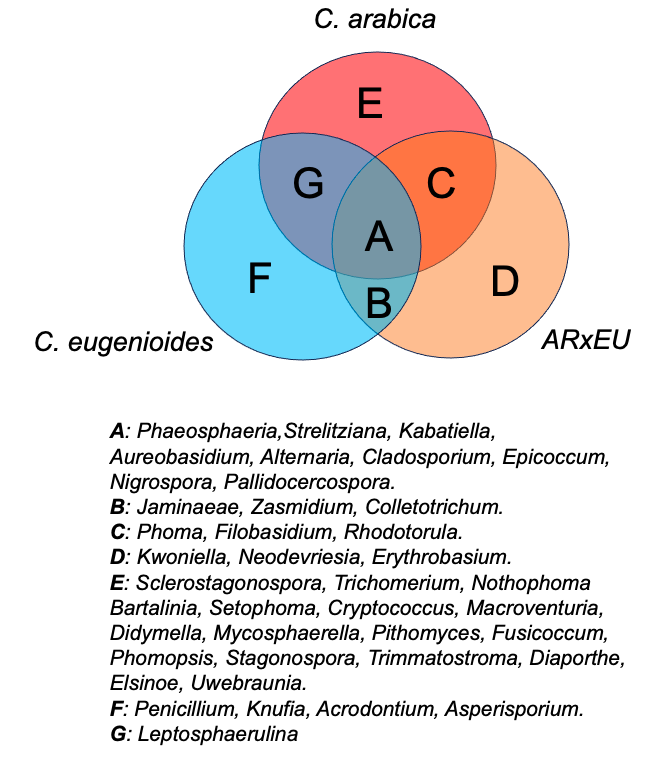


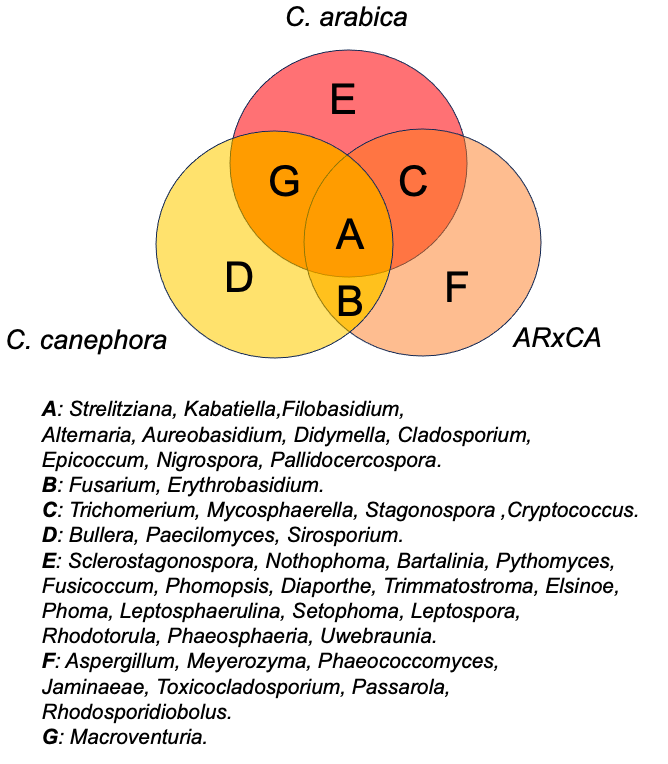


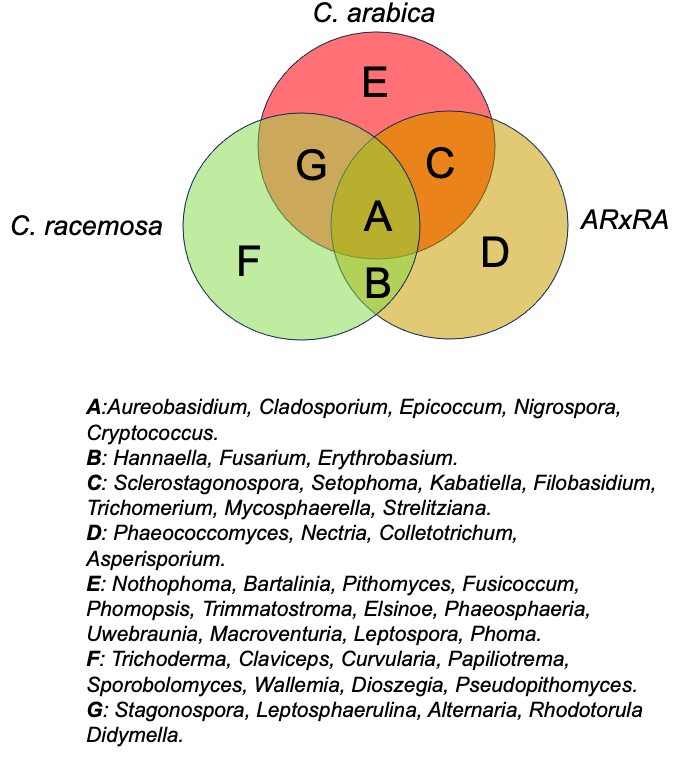


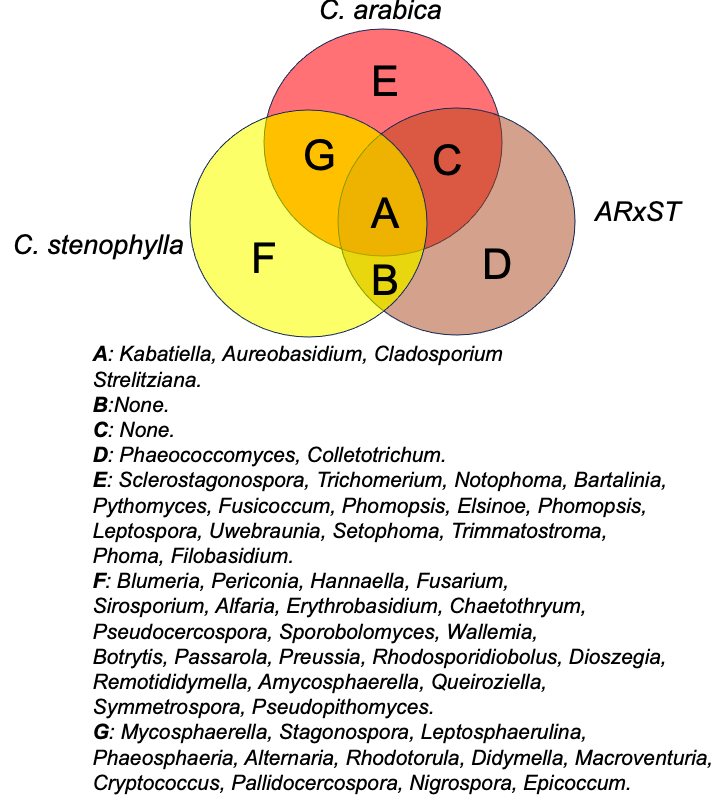


**Figure S4. Venn diagrams and list of fungal genera present in *Coffea* and hybrids**
